# Association between blood chromium and hepatic steatosis assessed by liver ultrasound transient elastography: National Health and Nutrition Examination Survey 2017–2020

**DOI:** 10.3389/fnut.2024.1307519

**Published:** 2024-04-24

**Authors:** Yingying Xiang, Ruonan Zhou, Ziwei Wang, Yingying Xue, Yue Cao, Lixuan Shen, Ziwei Zhu, Pingyuan Xu, Guowei Zhou, Wenbin Shang

**Affiliations:** ^1^Department of Endocrinology, Jiangsu Province Hospital of Chinese Medicine, The Affiliated Hospital of Nanjing University of Chinese Medicine, Nanjing, China; ^2^Key Laboratory for Metabolic Diseases in Chinese Medicine, Nanjing University of Chinese Medicine, Nanjing, China; ^3^Department of General Surgery, Jiangsu Province Hospital of Chinese Medicine, The Affiliated Hospital of Nanjing University of Chinese Medicine, Nanjing, China

**Keywords:** blood chromium, hepatic steatosis, fatty liver disease, controlled attenuated parameter, liver ultrasound transient elastography, NHANES

## Abstract

**Background:**

Hepatic steatosis is a significant pathological feature of fatty liver disease (FLD) which is widely spread with no effective treatment available. Previous studies suggest that chromium (Cr) intake reduces lipid deposition in the liver in animals. However, the connection between blood Cr and hepatic steatosis among humans remains inconclusive.

**Methods:**

Using the data from the National Health and Nutrition Examination Survey (NHANES) 2017–2020, we performed a cross-sectional analysis, including 4,926 participants. The controlled attenuation parameter (CAP) measured by the vibration controlled transient elastography (VCTE) was used to evaluate the degree of liver steatosis. Weighted univariate regression, multivariate linear regression, smooth fitting curves and subgroup analysis were used. In addition, we carried out trend tests, multiple interpolations, and interaction analyses to conduct sensitivity analyses.

**Results:**

After adjusting with various covariables, multivariate linear regression analysis demonstrated a significant negative correlation between blood Cr and CAP [β (95% CI) = −5.62 (−11.02, −0.21)]. The negative correlation between blood Cr and CAP was more significant in the males, 50–59 years, overweight, hypercholesterolemia, HDL-C ≥ 65 mg/dL, HbA1c (5.70–6.10 %), HOMA-IR (0.12–2.76), total bilirubin (0.30–0.40 mg/dL), ever alcohol consumption subjects. Of note, the relationships between blood Cr and CAP followed a U-shaped curve in the smokers and non-smokers, with blood Cr thresholds of 0.48, 0.69 μg/L, respectively.

**Conclusions:**

There is an independently negative correlation between blood Cr and hepatic steatosis in American. Our study provides clinical researchers with a new insight into the prospective prevention of hepatic steatosis.

## 1 Introduction

Fatty liver disease (FLD) is a prevalent and serious chronic liver disease worldwide, which potentially leads to steatohepatitis, fibrosis, cirrhosis and even death (1). Hepatic steatosis, defined as excessive storage of intrahepatic lipids, is the basis of FLD (2). The severity of hepatic steatosis can be evaluated by the controlled attenuation parameters (CAP) value using the vibration controlled transient elastography (VCTE) which has been proven to be a cost-effective, safe, and rapid non-invasive liver disease assessment tool (3). Given the lack of effective treatments for FLD, it is crucial to prevent the development of hepatic steatosis. Hepatic steatosis is closely related to disorders of glucolipid metabolism (4). Hence, improving the disorder of glucose and lipid metabolism is an important strategy for alleviating the severity of hepatic steatosis.

Organic chromium (Cr) is a trace dietary mineral, which participates in carbohydrate and lipid metabolism (5). Drugs reducing the concentration of blood Cr, such as doxepin and clozapine, were proven to worsen hepatic lipid deposition in obse rats, which indicate the potential role of blood Cr and hepatic steatosis (6, 7). A recent study observed an elevated blood and hepatic Cr as well as an ameliorated hepatic steatosis in BALB/c mice after Cr trichloride gavage (8). However, the relationship between blood Cr and hepatic steatosis in humans is still unknown.

With the worsen environmental pollution, inorganic Cr has increased in soil, which is widely recognized as a human carcinogen and an environmental pollutant (9). Seeing the contradictory effects of organic and inorganic Cr on human health, the relationship between blood Cr and various disease are controversial. The 2017–2020 US National Health and Nutrition Examination Survey (NHANES) assessed hepatic steatosis by VCTE and the level of blood Cr by inductively coupled plasma mass spectrometry (ICP-MS) in the U.S. population, which provided an opportunity to examine the importance of Cr and the relationship between blood Cr and hepatic steatosis.

## 2 Materials and methods

### 2.1 Study population and design

**Figure 1:**
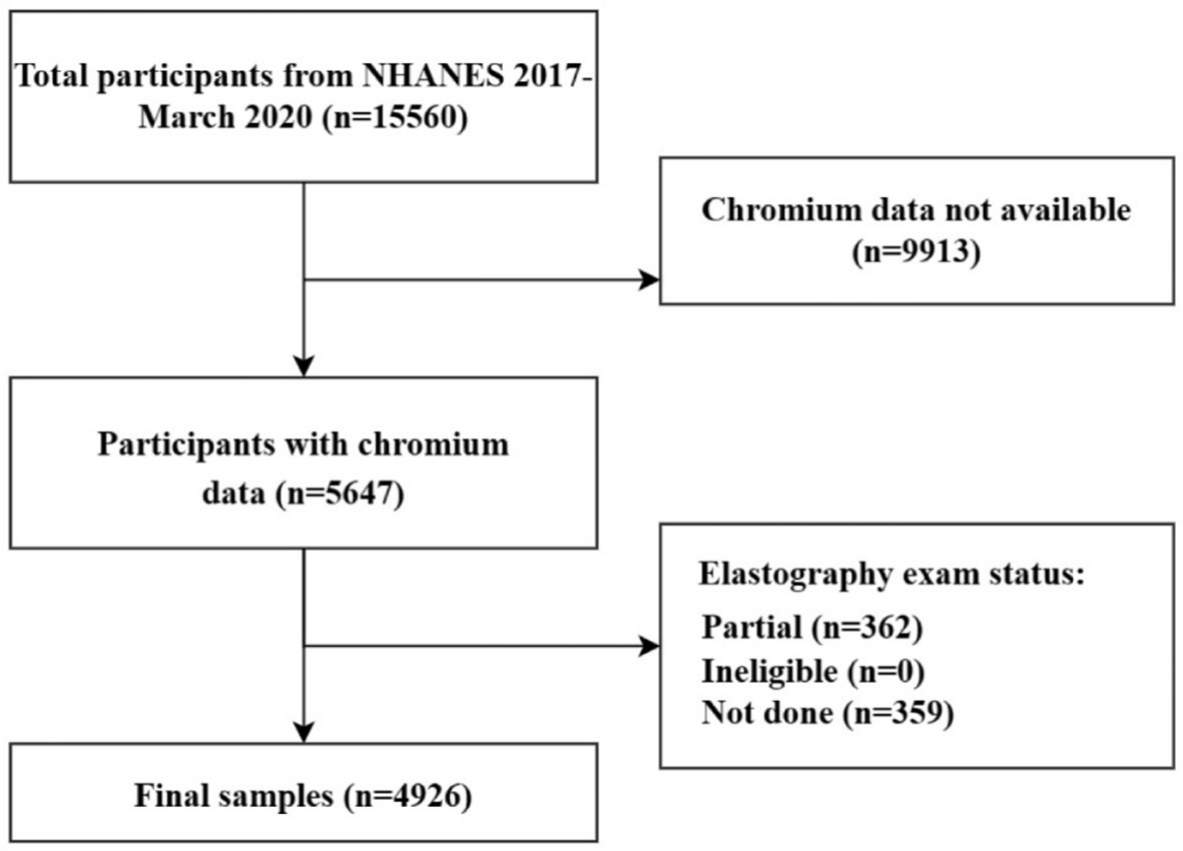
Flowchart of participant selection. NHANES, National Health and Nutrition Examination Survey.

Of 15,560 NHANES 2017–2020 participants who were included in the survey, 4,926 were eventually included in this study. This study established strict exclusion criteria, and 10,634 subjects were excluded based on the following criteria: (a) participants missing blood Cr data (*n* = 9,913); (b) participants missing and ineligible transient elastography data (*n* = 359); (c) participants partial exam transient elastography (*n* = 362; Figure 1).

### 2.2 Study variables

#### 2.2.1 Dependent variables: the controlled attenuation parameters

NHANES 2017–2020 used VCTE to estimate the degree of hepatic steatosis by measuring CAP. VCTE was performed by FibroScan model 502 V2 Touch (Echosens, Paris, France) equipped with a medium (M) or large (XL) probe. Only patients who satisfied the following conditions were considered to complete the assessment of hepatic steatosis: (a) fasting time ≥3 h; (b) a liver stiffness interquartile (IQR)/median <30%; (c) complete stiffness measures ≥10 times. CAP ≥285 dB/m was defined as hepatic steatosis (3).

#### 2.2.2 Independent variable: blood chromium levels

During the 2017–2020 US NHANES survey, blood samples were collected from participants aged 40+ years old at Mobile Examination Centers (MEC). These blood samples were then analyzed for blood Cr concentrations using ICP-MS. According to the *Centers for Disease Control and Prevention (CDC) Laboratory Procedure Manual*, the measurable range concentration of Cr was from 0.41 to 5,000 μg/L. An imputed fill value, which was calculated as the lower limit of detection (LLOD) divided by the square root of 2 [LLOD/sqrt [2]], was applied to analyze with results below LLOD. In addition, we identified individuals with blood Cr levels below 0.7 μg/L as Cr deficiency (10).

#### 2.2.3 Covariates

The socio-demographic (age, gender, race, education level, and family income-to-poverty ratio), anthropometry [liver stiffness measurement (LSM), body mass index (BMI), weight], biochemical indicators [alanine aminotransferase (ALT), aspartate aminotransferase (AST), gamma-glutamyl transferase (GGT), alkaline phosphatase (ALP), total bilirubin, urine albumin, total calcium, triglyceride (TG), total cholesterol (TC), low-density lipoprotein cholesterol (LDL-C), high-density lipoprotein cholesterol (HDL-C), blood cobalt, hs-CRP, HbA1c, insulin, HOMA-IR, HOMA-IS, and eGFR], health-related behaviors (smoking status, alcohol consumption) and comorbidities (diabetes, hypertension, hepatitis B, hepatitis C, and autoimmune hepatitis) data were used.

### 2.3 Statistical analysis

All continuous variables were statistically presented as mean ± SD (normal distribution) or median (quartile; skew distribution), and were compared by two sample *t*-test (normal distribution) or chi-square test (skew distribution) among groups, including age, BMI, weight, TG, TC, LDL-C, HDL-C, insulin, HbA1c, HOMA-IR, HOMA-IS, hs-CRP, ALT, AST, GGT, ALP, total bilirubin, blood Cr, blood cobalt, total calcium, urine albumin, eGFR, LSM, and CAP. Similarly, categorical variables were described using percentage or frequency, and compared by chi-square test among groups, including gender, race/ethnicity, education, family income-to-poverty threshold ratio (FITR), alcohol consumption, smoking status, hypertension, diabetes, hepatitis B, hepatitis C, and autoimmune hepatitis. Univariate and multivariate linear regression were used to explore the association between blood Cr and CAP. Three models were built in the multivariate test. Model I had no variables adjusted; Model II included age, gender, and race; Model III adjusted with all covariates mentioned above. Trend tests and subgroup analyses were also performed. The smooth curves through the analysis of generalized additive models (GAMs) were done to explore whether the non-linear relationship existed or not. Based on the piecewise regression model, we carried out a log-likelihood ratio test to examine the relationship and inflection point. Simultaneously, we used multiple imputations to fill miss continuous covariates, and dummy variables to present the categorical variables. Statistical significance was defined as having *p* < 0.05 (bilateral). We used the NHANES sample weights and accounted for unequal selection probabilities and non-response by applying standard methods. The statistical analyses were conducted by R version 4.2.0 (http://www.R-project.org) and EmpowerStats version 4.1 (http://www.empowerstat.com).

## 3 Results

### 3.1 Study population characteristics

A total of 4,926 participants were included in this study [Male: 2,448 (49.70%), Female: 2,478 (50.30%)]. The mean age of participants was 59.79 ± 11.63 years. The mean of CAP and the median (quartile) of blood Cr were 272.66 ± 60.21 dB/m, and 0.29 (0.29–0.29) μg/L, respectively. Significant statistical differences existed in CAP and blood Cr when participants were categorized by gender (*p* < 0.05; Supplementary Table 1). According to the cut-off point of CAP at 285 dB/m, the clinical characteristics of participants were demonstrated in Table 1. There were 2,064 in the hepatic steatosis group [Male: 1,124 (54.46%); Female: 940 (45.54%)], and 2,862 in the non-hepatic steatosis group [Male: 1,324 (46.26%); Female: 1,538 (53.74%)]. There was a significant statistical difference in blood Cr levels between the two groups (*p* < 0.05). There were also significance difference in gender, ethnicity, LSM, weight, BMI, ALT, AST, GGT, ALP, urine albumin, TG, HDL-C, blood cobalt, hs-CRP, HbA1c, HOMA-IS, eGFR, smoking status, diabetes, hypertension, hepatitis B, and hepatitis C between the groups (*p* < 0.05), and we take these as covariates in the following study.

**Table 1:** Baseline characteristics of the study population based on the controlled attenuated parameter (CAP).

**Characteristic**	**Total (*n* = 4,926)**	**Non-hepatic steatosis (CAP <285 dB/m, *n* = 2,862)**	**Hepatic steatosis (CAP ≥285 dB/m, *n* = 2,064)**	***p*-value**
**Socio-demographic characteristics**				
**Gender**				<0.01
Man	2,448 (49.70%)	1,324 (46.26%)	1,124 (54.46%)	
Woman	2,478 (50.30%)	1,538 (53.74%)	940 (45.54%)	
Age (years)	59.79 ± 11.63	60.05 ± 11.97	59.43 ± 11.14	0.116
**Ethnicity**				<0.01
Hispanic	1,047 (21.25%)	523 (18.27%)	524 (25.39%)	
Non-Hispanic White	1,787 (36.28%)	997 (34.84%)	790 (38.28%)	
Non-Hispanic Black	1,278 (25.94%)	838 (29.28%)	440 (21.32%)	
Non-Hispanic Asian	597 (12.12%)	373 (13.03%)	224 (10.85%)	
Other race	217 (4.41%)	131 (4.58%)	86 (4.17%)	
**Education**				0.73
Less than high school	983 (19.96%)	568 (19.85%)	415 (20.11%)	
High school	1,174 (23.83%)	673 (23.52%)	501 (24.27%)	
More than high school	2,760 (56.03%)	1,617 (56.50%)	1,143 (55.38%)	
Not recorded	9 (0.18%)	4 (0.14%)	5 (0.24%)	
**FITR (%)**				0.73
<1.0	712 (14.45%)	414 (14.47%)	298 (14.44%)	
1.0 to <2.0	1,103 (22.39%)	635 (22.19%)	468 (22.67%)	
2.0 to <3.0	677 (13.74%)	401 (14.01%)	276 (13.37%)	
3.0 to <5.0	877 (17.80%)	491 (17.16%)	386 (18.70%)	
≥5	901 (18.29%)	534 (18.66%)	367 (17.78%)	
Not recorded	656 (13.32%)	387 (13.52%)	269 (13.03%)	
**Physical examinations**				
LSM (kPa)	5.10 (4.20–6.40)	4.80 (4.00–5.80)	5.80 (4.60–7.30)	<0.01
CAP (dB/m)	272.66 ± 60.21	231.24 ± 37.04	330.09 ± 32.74	<0.01
Weight (kg)	82.88 ± 21.01	75.75 ± 17.59	92.72 ± 21.38	<0.01
BMI (kg/m^2^)	29.93 ± 6.78	27.57 ± 5.72	33.19 ± 6.77	<0.01
**Biochemical indicators**				
ALT (U/L)	18.00 (13.00–26.00)	16.00 (12.00–23.00)	21.00 (15.00–30.00)	<0.01
AST (U/L)	19.00 (16.00–24.00)	19.00 (16.00–23.00)	20.00 (16.00–25.00)	<0.01
GGT (IU/L)	22.00 (15.25–34.00)	20.00 (14.00–30.00)	26.00 (19.00–41.00)	<0.01
ALP (IU/L)	80.13 ± 25.72	78.32 ± 25.42	82.61 ± 25.93	<0.01
Total bilirubin (mg/dL)	0.40 (0.30–0.60)	0.40 (0.30–0.60)	0.40 (0.30–0.60)	0.21
Urine albumin (ug/mL)	9.40 (4.70–20.55)	8.60 (4.30–19.00)	10.50 (5.38–23.83)	<0.01
Total calcium (mg/dL)	9.28 ± 0.39	9.27 ± 0.39	9.28 ± 0.39	0.13
TG (mg/dL)	97.00 (67.00–139.00)	83.00 (60.00–118.00)	117.00 (85.00–164.00)	<0.01
TC (mg/dL)	190.07 ± 42.21	190.87 ± 42.02	188.97 ± 42.47	0.12
LDL-C (mg/dL)	111.42 ± 36.97	112.39 ± 36.56	110.05 ± 37.51	0.13
HDL-C (mg/dL)	54.09 ± 16.41	57.78 ± 16.86	49.01 ± 14.30	<0.01
Blood cobalt (ug/L)	0.14 (0.11–0.18)	0.14 (0.11–0.19)	0.14 (0.10–0.17)	<0.01
Blood chromium (μg/L)	0.29 (0.29–0.29)	0.29 (0.29–0.29)	0.29 (0.29–0.29)	<0.01
hs-CRP (mg/L)	2.04 (0.91–4.44)	1.60 (0.75–3.55)	2.85 (1.29–5.75)	<0.01
HbA1c (%)	6.04 ± 1.18	5.82 ± 0.96	6.36 ± 1.37	<0.01
Insulin (μU/mL)	10.11 (6.30–16.39)	7.84 (5.15–11.84)	14.92 (9.48–22.63)	0.55
HOMA-IR	2.76 (1.66–4.88)	2.06 (1.32–3.31)	4.44 (2.68–7.16)	0.35
HOMA-IS	0.11 (0.07–0.18)	0.09 (0.06–0.14)	0.16 (0.10–0.24)	<0.05
eGFR (ml/min/1.73 m^2^)	79.87 ± 31.84	78.33 ± 31.73	82.01 ± 31.88	<0.01
**Lifestyle factors**				
**Alcohol consumption**				0.28
Never	1,337 (27.14%)	777 (27.15%)	560 (27.13%)	
≤ 3 times/month	2,028 (41.17%)	1,147 (40.08%)	881 (42.68%)	
Once a week	306 (6.21%)	178 (6.22%)	128 (6.20%)	
≥2 times/week	583 (11.84%)	352 (12.30%)	231 (11.19%)	
Not recorded	672 (13.64%)	408 (14.26%)	264 (12.79%)	
**Smoking status**				<0.01
Yes	829 (16.83%)	521 (18.20%)	308 (14.92%)	
No	1,377 (27.95%)	740 (25.86%)	637 (30.86%)	
Not recorded	2,720 (55.22%)	1,601 (55.94%)	1,119 (54.22%)	
**Comorbidities**				
**Diabetes**				<0.01
Normal	1,944 (39.46%)	1,375 (48.04%)	569 (27.57%)	
Prediabetes	1,767 (35.87%)	1,013 (35.39%)	754 (36.53%)	
Diabetes	1,215 (24.67%)	474 (16.56%)	741 (35.90%)	
**Hypertension**				<0.01
Normal	2,513 (51.02%)	1,597 (55.80%)	916 (44.38%)	
Hypertension	2,406 (48.84%)	1,261 (44.06%)	1,145 (55.47%)	
Not recorded	7 (0.14%)	4 (0.14%)	3 (0.15%)	
**Hepatitis B**				<0.01
Normal	4,403 (89.38%)	2,515 (87.88%)	1,888 (91.47%)	
Hepatitis B	521 (10.58%)	346 (12.09%)	175 (8.48%)	
Not recorded	2 (0.04%)	1 (0.03%)	1 (0.05%)	
**Hepatitis C**				<0.01
Normal	4,742 (96.26%)	2,732 (95.46%)	2,010 (97.38%)	
Hepatitis C	183 (3.71%)	130 (4.54%)	53 (2.57%)	
Not recorded	1 (0.02%)	0 (0.00%)	1 (0.05%)	
**Autoimmune hepatitis**				0.94
Autoimmune hepatitis	14 (0.28%)	8 (0.28%)	6 (0.29%)	
Not recorded	4,912 (99.72%)	2,854 (99.72%)	2,058 (99.71%)	

LDL-C, low-density lipoprotein cholesterol; HDL-C, high-density lipoprotein cholesterol; TC, total cholesterol; TG, triglyceride; HbA1c, glycated hemoglobin; hs-CRP, high-sensitivity C-reactive protein; ALT, alanine aminotransferase; ALP, alkaline phosphatase; AST, aspartate aminotransferase; GGT, gamma-glutamyl transferase; BMI, body mass index; LSM, liver stiffness measure; CAP, controlled attenuation parameter; FITR, Family income-to-poverty threshold ratio.

**Figure 2:**
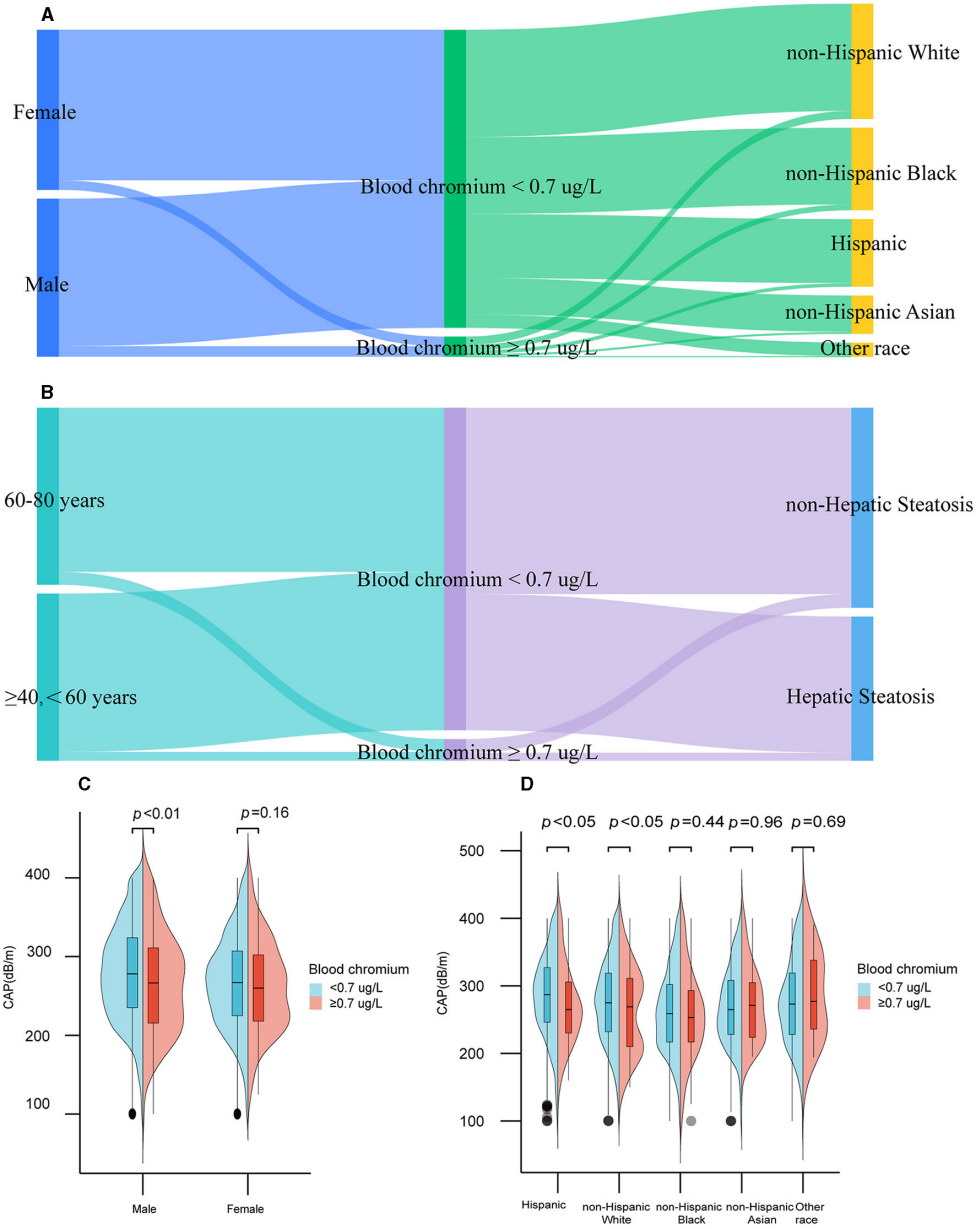
Distribution of blood chromium by gender, ethnicity, age, and hepatic steatosis among participants in the United States, 2017–2020. Distribution of blood chromium by gender, ethnicity **(A)**, age, and hepatic steatosis **(B)**. CAP at different gender **(C)** and ethnicity **(D)** among blood chromium deficiency or normal participants.

As shown in Figures 2A, B and Supplementary Table 2, Cr deficiency was widespread in Americans aged 40+ [Cr deficiency vs. normal: 4,615 (93.69%) vs. 311(6.31%)]. Besides, males with blood Cr deficiency had higher CAP compared to those with normal blood Cr levels (*p* < 0.05; Figure 2C). As shown in Figure 2D, Hispanic population and non-Hispanic White population with blood Cr deficiency had the higher level of CAP (*p* < 0.05).

### 3.2 Associations between other covariates and hepatic steatosis

**Figure 3:**
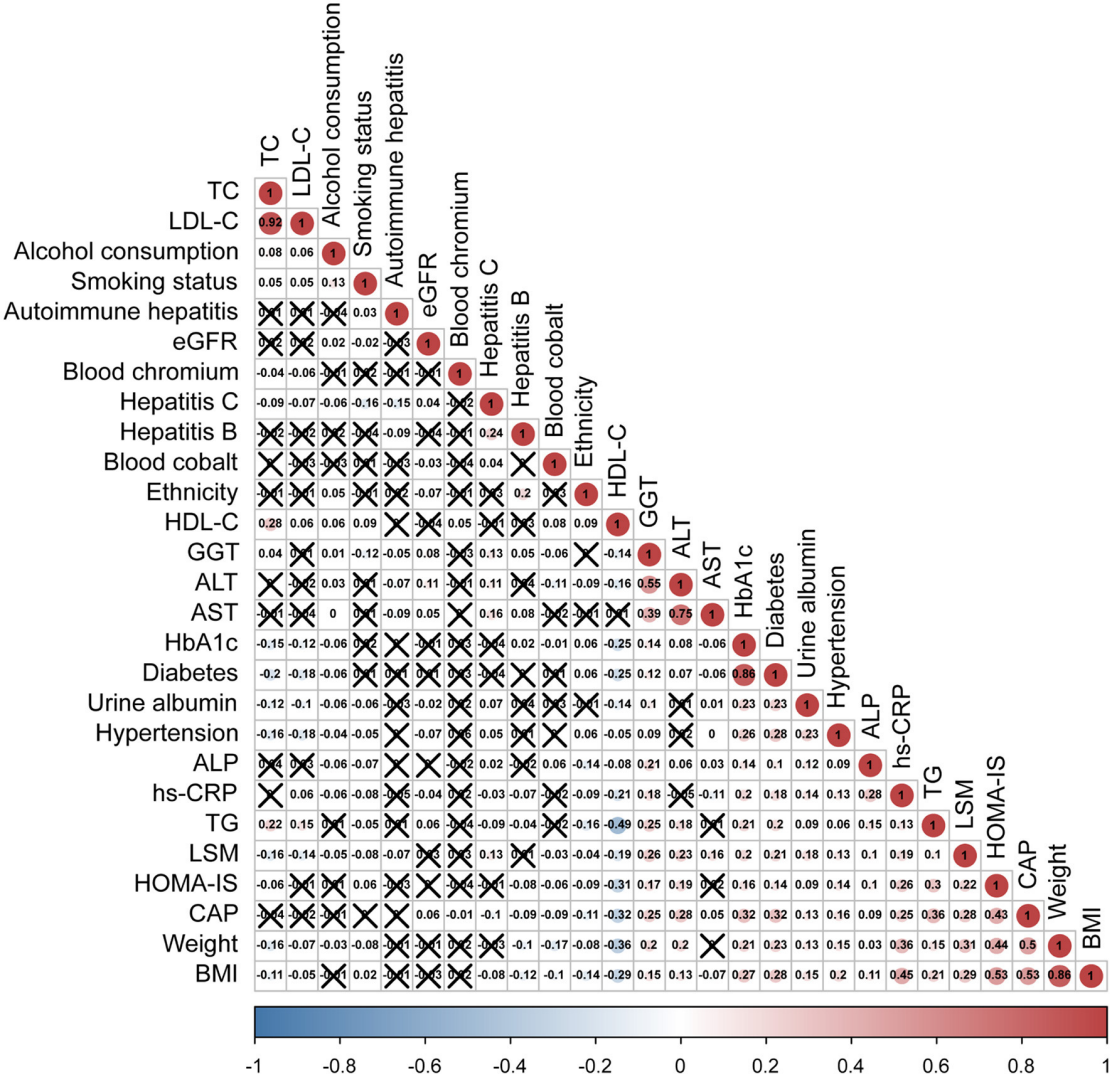
Spearman's rank correlation coefficient plot. With the decrease of Spearman's r, the blue in the figure deepens, which means the negative correlation is stronger; with the increase of Spearman's *r*, the red in the figure deepens, which means that the positive correlation is stronger. LDL-C, low-density lipoprotein cholesterol; HDL-C, high-density lipoprotein cholesterol; TC, total cholesterol; TG, triglyceride; HbA1c, glycated hemoglobin; hs-CRP, high-sensitivity C-reactive protein; ALT, alanine aminotransferase; ALP, alkaline phosphatase; AST, aspartate aminotransferase; GGT, gamma-glutamyl transferase; BMI, body mass index; LSM, liver stiffness measure; CAP, controlled attenuation parameter.

To find the covariables which affected the degree of hepatic steatosis, we used the Spearman's correlation to analyze the between-group variation and other hepatic steatosis related covariables. As represented in Figure 3, CAP levels were not only correlated with blood Cr, but also with gender, ethnicity, LSM, weight, BMI, TG, HDL-C, HbA1c, HOMA-IS, ALT, AST, GGT, ALP, blood cobalt, urine albumin, eGFR, hs-CRP, diabetes, hypertension, hepatitis C, and hepatitis B (*p* < 0.05).

Next, univariate regression analysis was used to observe the degree of association between CAP and the above related covariates in the US population. As shown in Table 2, blood Cr had a significant negative correlation with the CAP levels [β (95% CI) = −5.19 (−8.93, −1.46)] (*p* < 0.01). Compared with males, females were less likely to develop hepatic steatosis [β (95% CI) = −12.20 (−15.54, −8.85)] (*p* < 0.01). Among different ethnicity, with Hispanic as the reference, non-Hispanic black had a lower degree of hepatic steatosis [β (95% CI) = −21.88 (−26.75, −17.00)] (*p* < 0.01). Hepatic steatosis had positive correlations with LSM, weight, BMI, ALT, AST, GGT, ALP, TG, hs-CRP, HbA1c, insulin, HOMA-IR, HOMA-IS, eGFR, smoking status, diabetes, and hypertension. Conversely, hepatic steatosis showed negative correlations with age, HDL-C, hepatitis C, and hepatitis B (*p* < 0.05).

**Table 2:** Univariate regression analysis for the controlled attenuation parameter (CAP).

**Covariates**	**β (95% CI) *p-*value**	**Covariates**	**β (95% CI) *p-*value**
**Gender**		**Hepatitis C**	
Man	Reference	Normal	Reference
Woman	−12.20 (−15.54, −8.85) <0.01	Hepatitis C	−19.93 (−28.80, −11.05) <0.01
**Ethnicity**		Not recorded	61.61 (−56.18, 179.41) 0.31
Hispanic	Reference	LSM (kPa)	1.93 (1.58, 2.28) <0.01
Non–Hispanic White	−9.05 (−13.60, −4.49) <0.01	Weight (kg)	1.39 (1.32, 1.46) <0.01
Non–Hispanic Black	−21.88 (−26.75, −17.00) <0.01	BMI (kg/m^2^)	4.37 (4.16, 4.59) <0.01
Non–Hispanic Asian	−15.39 (−21.39, −9.38) <0.01	Blood chromium (μg/L)	−5.19 (−8.93, −1.46) <0.01
Other race	−7.80 (−16.53, 0.93) 0.08	ALT (U/L)	0.55 (0.46, 0.64) <0.01
**Diabetes**		AST (U/L)	0.16 (0.05, 0.27) <0.05
Normal	Reference	GGT (IU/L)	0.13 (0.09, 0.16) <0.01
Prediabetes	19.11 (15.40, 22.82) <0.01	ALP (IU/L)	0.19 (0.12, 0.25) <0.01
Diabetes	45.26 (41.13, 49.38) <0.01	Urine albumin (ug/mL)	−0.00 (−0.01, 0.00) 0.10
**Hypertension**		TG (mg/dL)	0.13 (0.11, 0.16) <0.01
Normal	Reference	HDL–C (mg/dL)	−1.11 (−1.21, −1.01) <0.01
Hypertension	16.64 (13.30, 19.97) <0.01	Blood cobalt (ug/L)	−1.70 (−3.96, 0.56) 0.14
Not recorded	25.50 (−18.74, 69.75) 0.26	HbA1c (%)	12.89 (11.51, 14.27) <0.01
**Hepatitis B**		hs–CRP(mg/L)	0.53 (0.34, 0.72) <0.01
Normal	Reference	HOMA–IS	41.94 (32.88, 50.99) <0.01
Hepatitis B	−12.88 (−18.33, −7.42) <0.01	eGFR	0.12 (0.06, 0.17) <0.01
Not recorded	−21.53 (−104.82, 61.76) 0.61		

LDL–C, low–density lipoprotein cholesterol; HDL–C, high–density lipoprotein cholesterol; TC, total Cholesterol; TG, triglyceride; HbA1c, glycated hemoglobin; hs–CRP, high–sensitivity C-reactive protein; ALT, alanine aminotransferase; ALP, alkaline phosphatase; AST, aspartate aminotransferase; GGT, gamma-glutamyl transferase; BMI, body mass index; LSM, liver stiffness measure; CAP, controlled attenuation parameter.

### 3.3 Weighted multivariate linear regression between blood chromium (Cr) and the controlled attenuation parameter (CAP)

Weighted multivariate linear regression analysis was conducted to evaluate the relationship between blood Cr and CAP in participants. Based on the results of the correlation analysis and the clinical significance, we adjusted the model with various covariates (age, gender, ethnicity, education, family income to-poverty threshold ratio, alcohol consumption, smoking status, BMI, weight, TG, TC, LDL-C, HDL-C, insulin, HbA1c, HOMA-IR, HOMA-IS, hs-CRP, ALT, AST, GGT, ALP, total bilirubin, blood cobalt, total calcium, urine albumin, eGFR, hypertension, diabetes, hepatitis B, hepatitis C, autoimmune hepatitis, and LSM). As demonstrated in Table 3, blood Cr had a negative correlation with the value of CAP. The negative correlation between blood Cr and CAP was present in the unadjusted model [β (95% CI) = −5.19 (−8.93, −1.46)], model II [β (95% CI) = −4.83 (−8.52, −1.14)], and model III [β (95% CI) = −5.62 (−11.02, −0.21)] (*p* < 0.05). In addition, to ensure the stability of the results, blood Cr was categorized into three classes according to clinical significance, and trend test was carried out in this study. With blood Cr <0.41 g/L as reference, model I–III showed a negative correlation between blood Cr level and CAP (all *p* for trend <0.05), which indicated that there was a stable and negative correlation between blood Cr level and the CAP. After the interpolation, the negative correlation also existed (Supplementary Table 3).

**Table 3:** Multivariate linear regression between blood chromium and the controlled attenuation parameter (CAP).

	**Model I**		**Model II**		**Model III**	
	**β (95% CI)**	***p*-value**	**β (95% CI)**	***p*-value**	**β (95% CI)**	***p*-value**
Blood chromium	−5.19 (−8.93, −1.46)	<0.01	−4.83 (−8.52, −1.14)	<0.05	−5.62 (−11.02, −0.21)	<0.05
**Blood chromium categories**						
<0.41 μg/L	Reference		Reference		Reference	
≥ 0.41, <0.7 μg/L	−4.78(−10.11, 0.55)	0.08	−3.81(−9.09, 1.46)	0.16	0.41(−5.86, 6.68)	0.90
≥ 0.7 μg/L	−10.05 (−16.98, −3.11)	<0.01	−9.32 (−16.18, −2.46)	<0.01	−10.12 (−19.29, −0.96)	<0.05
*p* for trend	<0.01		<0.01		<0.05	

Model I: no covariates were adjusted.

Model II: age, gender, and race/ethnicity were adjusted.

Model III: age, gender, race/ethnicity, education, family income-to-poverty threshold ratio, alcohol consumption, smoking status, BMI, weight, TG, TC, LDL-C, HDL-C, insulin, HbA1c, HOMA-IR, HOMA-IS, hs-CRP, ALT, AST, GGT, ALP, total bilirubin, blood cobalt, total calcium, urine albumin, eGFR, hypertension, diabetes, hepatitis B, hepatitis C, autoimmune hepatitis, and LSM were adjusted.

### 3.4 Subgroup analysis

**Figure 4:**
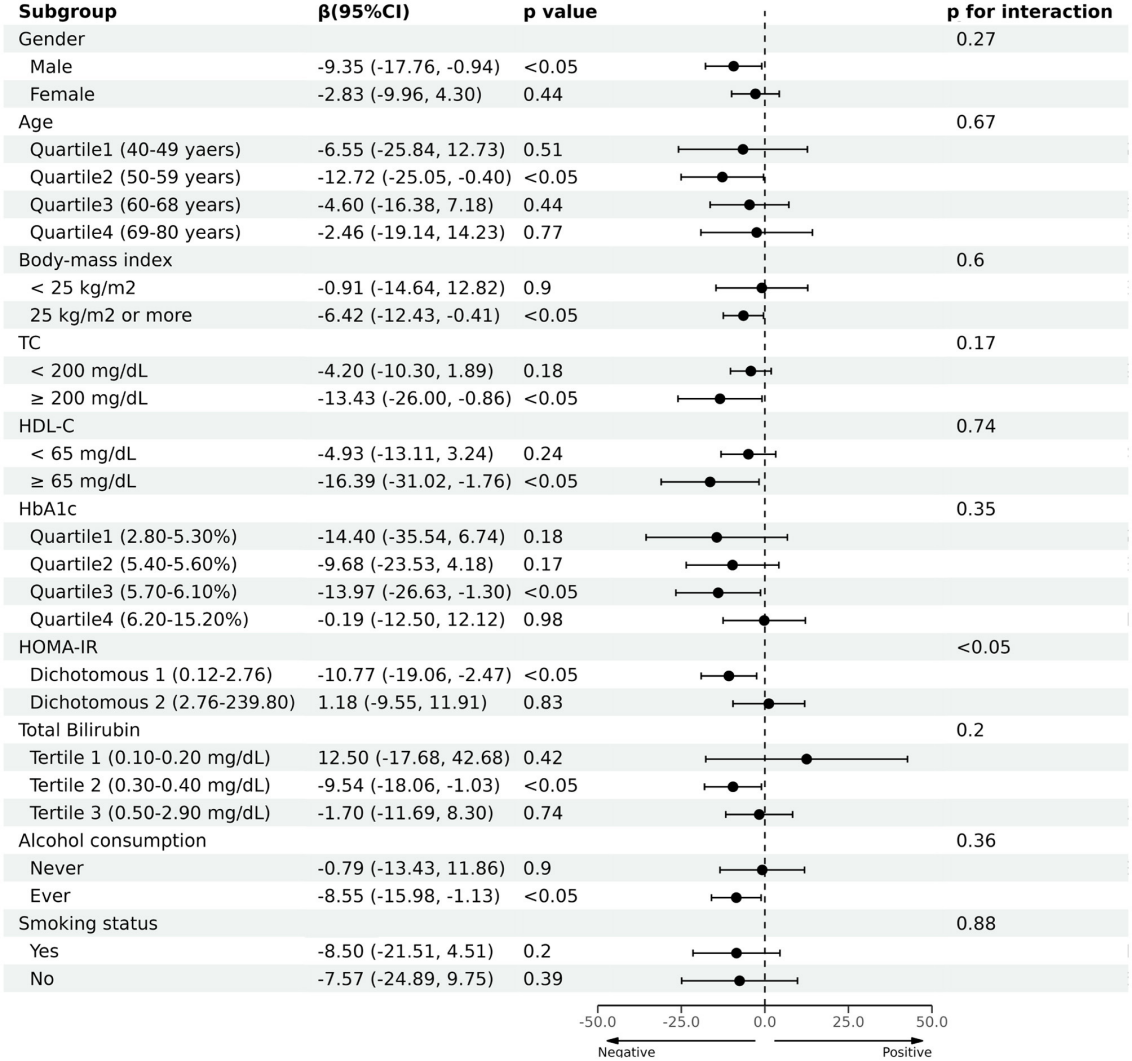
Association between blood chromium and the controlled attenuation parameter (CAP) in prespecified and exploratory subgroups.

Next, we conducted subgroup analysis by dividing participants into groups by gender, age, BMI, TC, HDL-C, HbA1c, HOMA-IR, total bilirubin, alcohol consumption, and smoking status, respectively. As shown in Figure 4, it was noted that blood Cr had a negative correlation with CAP in the male [β (95% CI) = −9.35 (−17.76, −0.94)], age quartile 2 (50–59 years) [β (95% CI) = −12.72 (−25.05, −0.40)], overweight [β (95% CI) = −6.42 (−12.43, −0.42)], hypercholesterolemia [β (95% CI) = −13.43 (−26.00, −0.86)], HDL-C ≥ 65 mg/dL [β (95% CI) = −16.39 (−31.02, −1.76)], HbA1c quartile 3 (5.70–6.10%) [β (95% CI) = −13.97 (−26.63, −1.30)], HOMA-IR dichotomous 1 (0.12–2.76) [β (95% CI) = −11.38 (−19.68, −3.09)], total bilirubin tertile 2 (0.30–0.40 mg/dL) [β (95% CI) = −9.54 (−18.06, −1.03)] and ever alcohol consumption [β (95% CI) = −8.55 (−15.98, −1.13)] (*p* < 0.05). Furthermore, the negative correlation between blood Cr and CAP was also significant in (*p* < 0.05). The smoking status didn't affect the presence of the relationship between CAP and blood Cr (*p* > 0.05). Moreover, we conducted an interaction test, which found that negative correlation between blood Cr and CAP was significantly modified by HOMA-IR (*p* for interaction <0.05).

### 3.5 Dose-response relationship between blood chromium and the controlled attenuation parameter (CAP)

**Figure 5:**
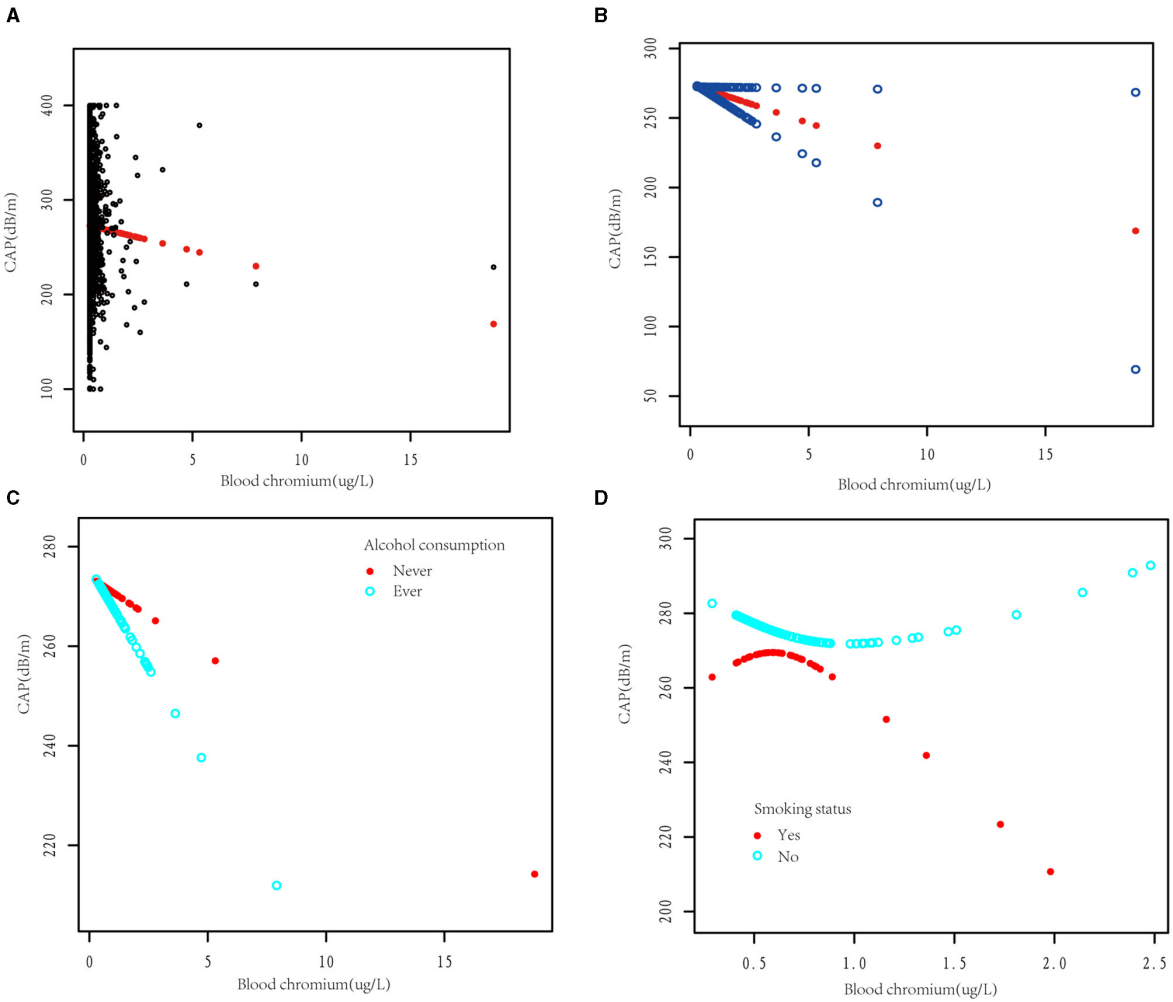
The dose-response association between blood chromium and the controlled attenuation parameter (CAP). **(A)** Each black point represents a sample subject. **(B)** The solid red line represents the smooth curve fit between variables. Blue bands represent the 95% confidence interval from the fit. The dose-response relationship between blood chromium and CAP was stratified by alcohol consumption **(C)** and smoking status **(D)**. Adjusted for age, gender, race/ethnicity, education, family income-to-poverty threshold ratio, alcohol consumption, smoking status, BMI, weight, TG, TC, LDL-C, HDL-C, insulin, HbA1c, HOMA-IR, HOMA-IS, hs-CRP, ALT, AST, GGT, ALP, total bilirubin, blood cobalt, total calcium, urine albumin, eGFR, hypertension, diabetes, hepatitis B, hepatitis C, autoimmune hepatitis, and LSM except the stratification variable.

The smooth curve fitting diagrams were drawn to visually estimate the dose-response relationship between blood Cr and CAP by GAMs. As shown in Figures 5A, B, the relationship between the blood Cr and CAP was linear after adjusting with all covariates [β (95% CI) = −5.62 (−11.02, −0.21)].

Due to alcohol consumption and smoking being common unhealthy lifestyle habits, we subsequently investigated whether alcohol consumption and smoking status would affect the dose relationship between blood Cr and CAP. After dividing the participants according to alcohol consumption, we observed that blood Cr showed a negative linear relationship with CAP in both alcohol consumption and non-alcohol consumption group (Figure 5C). Different with the results above, while dividing the participants with smoking status, the relationship between blood Cr and CAP showed U-shape curves in both somking and non-smoking groups. In smoking participants with blood Cr above 0.48 μg/L and non-smoking participants with blood Cr below 0.69 μg/L, the blood Cr showed a negative correlation with CAP (*p* < 0.05); while in smoking participants with blood Cr below 0.48 μg/L and non-smoking participants with blood Cr above 0.69 μg/L, the negative relationships between blood Cr and CAP were disturbed (*p* for log likelihood ratio test <0.05; Figure 5D and Table 4).

**Table 4:** Threshold effect analysis of blood chromium on the controlled attenuation parameter (CAP) stratified by smoking status using two-piecewise linear regression model.

	**Smoker**	**Non-smoker**
	**Adjusted β (95% CI)**	**Adjusted β (95% CI)**
	***p*-value**	***p*-value**
Inflection point	0.48	0.69
<Inflection point	86.35 (−14.85, 187.55) 0.10	−42.74 (−79.63, −5.85) <0.05
>Inflection point	−55.26 (−104.36, −6.15) <0.05	18.88 (−11.11, 48.86) 0.22
*p* for log likelihood ratio test	<0.05	<0.05

Adjusted for age, gender, race/ethnicity, education, family income-to-poverty threshold ratio, alcohol consumption, smoking status, BMI, weight, TG, TC, LDL-C, HDL-C, insulin, HbA1c, HOMA-IR, HOMA-IS, hs-CRP, ALT, AST, GGT, ALP, total bilirubin, blood cobalt, total calcium, urine albumin, eGFR, hypertension, diabetes, hepatitis B, hepatitis C, autoimmune hepatitis, and LSM except for the stratification variable.

## 4 Discussion

Our results suggest that blood Cr level is associated with hepatic steatosis in Americans. FLD is a wide spread chronic liver disease, and at the same time, one of the leading causes of death (11). In this study, we found that an elevation of 1 μg/L in blood Cr is corresponded with a decrease in CAP by −5.62 (−11.02, −0.21) dB/m, suggesting that the effect of intervening blood Cr may be a potential therapeutic strategy of FLD.

To our current knowledge, this research is the first to present the association between blood Cr level and hepatic steatosis in a large population. In earlier studies, researchers found that patients provided with an oral dose of 400 μg of Cr picolinate (CrPic) for 3 months showed the same degree of hepatic steatosis as the placebo group (12). However, dietary Cr intake does not reflect Cr absorption, and blood Cr level is a better indicator of Cr metabolism and distribution in the body, which was not investigated in this study (13). Furthermore, this study was also limited by its small sample size. In line with our results, another randomized controlled clinical trial reported that 1,000 μg CrPic daily intake for 24 weeks resulted in an increase in blood Cr level as well as a significant reduction in hepatic lipid deposition among type 2 diabetes mellitus (T2DM) insulin sensitivity responders (14). These findings remind us that insulin sensitivity may play a role in the interactivities between Cr and hepatic steatosis.

The underlying mechanisms of the negative association between blood Cr and hepatic steatosis may involves the alterations in insulin sensitivity, immunity, oxidative stress, gluco-lipid metabolism and gut microbiota by Cr. Impaired insulin sensitivity disrupts the balance of glucose and lipid metabolism, increasing the risk of hepatic steatosis (15), and trivalent Cr, as a co-factor to activate the insulin receptor, improves insulin action in insulin-sensitive tissues, such as adipose, skeletal muscle, liver tissue, etc. (16, 17). In adipose tissue, Cr supplementation upregulates peroxisome proliferator-activated receptor-γ (PPARγ) expression in T2DM rats (18). The activation of PPARγ pathway reduces the free fatty acid (FFA)-induced damage and restoring insulin sensitivity in peripheral tissues (19). In skeletal muscle, Cr increases the activity of Adenosine 5′-monophosphate (AMP)-activated protein kinase (AMPK) and upregulates the gene expression of insulin receptor (IR), glucose transporter 4 (GLUT4), and uncoupling protein-3 (UCP-3), improving insulin sensitivity and promoting the glucose transport (20, 21). In liver, Cr supplementation inhibited the expression of insulin degrading enzyme (IDE) in liver of KKAy mice and HepG2 cells, promoting insulin signal transduction (22). Moreover, Cr supplementation increased the p-IRS-1, GLUT2, and GLUT4 expression in the liver of various animal models of metabolic disorders, such as NAFLD, obesity, T2DM and metabolic syndrome, and thus lowering the hepatic TG levels of these models (23–27).

Cr has also been proven to regulate immune response and alleviate liver inflammation in animal models of obesity, metabolic syndrome, T2DM, and FLD. Cr supplementation decreased the expression of liver nuclear factor kappa-B (NF-κB) p65, and lowered plasma levels of C-reactive protein (CRP), monocyte chemoattractant protein-1 (MCP-1), and intercellular cell adhesion molecule-1 (ICAM-1) in animal models of obesity, metabolic syndrome and T2DM. Furthermore, Cr supplementation downregulates CD68 and myelo-peroxidase (MPO) protein expression, reduces levels of pro-inflammatory cytokines in serum, and increases the secretion of the anti-inflammatory cytokine in hepatic steatosis animal models (8, 23, 24, 27, 28).

Cr also plays a positive role in affecting oxidative stress. *In vitro* experiments, Cr supplementation effectively protects LO2 cells from H_2_${\text{O}}_{2}^{-}$ induced oxidative damage, maintaining mitochondrial integrity and improving cell viability (29). In NAFLD KK/HlJ mice, Cr supplementation was found to reduce hepatic malondialdehyde (MDA) levels, increase liver Cu/Zn-SOD, catalase (CAT), and glutathione peroxidase (GPx) levels, and inhibit oxidative stress (24). In aged diabetic Zucker fatty rats and Spontaneously hypertensive rats, supplementation of Cr compounds reduced the hepatic and renal lipid peroxidation and DNA fragmentation (30, 31).

Cr affects energy metabolism and suppresses hepatic lipid deposition by modulating glucolipid metabolism-related enzymes. Cr supplementation inhibits the activities of α-amylase and α-glucosidase, exerting a beneficial effect on postprandial blood glucose (28, 29). In animal models of metabolic syndrome and T2DM, Cr supplementation inhibited hepatic gluconeogenesis by decreasing mRNA expression of fructose 1,6-bisphosphatase (FBPase), phosphoenolpyruvate carboxykinase (PEPCK), and glucose-6-phosphatase (G6Pase) and promoted hepatic glycogen synthesis by upregulating glucokinase (Gk) mRNA expression in the liver (32–34). In addition, Cr reduced the expression of CD36 in liver tissue and SMMC-7721 cells, thus decreasing the uptake of exogenous fatty acids, while increasing the levels of liver type fatty acid binding protein (L-FABP) mRNA in liver tissue, promoting fatty acid transport across cell membranes and into mitochondria, which enhanced hepatic fatty acid utilization (8, 33–36). In animal models of NAFLD and metabolic syndrome, Cr supplementation downregulated the expression of sterol regulatory element-binding protein-1 (SREBP-1) mRNA, which is a key transcription factors involved in the synthesis of cholesterol and fatty acids (28, 33, 37). In animal models of NAFLD and non-alcoholic fatty liver hepatitis (NASH), Cr downregulated the mRNA expression of diacylglycerolacyltransferase (DGAT)-1, DGAT-2, and fatty acid synthase (FASN), while suppressed protein expression of perilipin-2 (PLIN-2), consequently lessening the storage of TG in liver tissue (28). Furthermore, Cr enhances cholesterol metabolism. In Kunming mice with metabolic syndrome, Cr supplementation promoted cholesterol transport and metabolism by upregulating the mRNA expression of apolipoprotein E (ApoE) and low-density lipoprotein receptor (LDLR) and suppressed cholesterol synthesis by upregulating cytochrome P450 7A1 (CYP7A1) mRNA expression (32, 33). Finally, recent studies suggest that Cr supplementation significantly alteres the gut microbiome in animal models of T2DM and metabolic syndrome, leading to the improvement of glucose and lipid metabolism and the inhibition of liver lipid accumulation by the “gut-liver axis” (32–34).

By dividing the participants according to smoking status, the correlation between blood Cr and CAP were disturbed in smoking participants. This phenomenon may be related to Cr deposition in the lung caused by smoking. Cr not only distributes in blood, but also deposites in lung, and the pulmonary Cr concentration increases with the elevation of the smoking time (38, 39). Noteworthily, smoking primarily induces ectopic deposition of lipids in the liver through the induction of inflammation, oxidative stress, gut microbiota dysbiosis, disturbances in glucose and lipid metabolism and insulin resistance (40, 41). Additionally, the pulmonary Cr may alleviate the toxic effects of nicotine on liver by multiple ways (24, 28, 34, 42). However, the specific mechanism by which nicotine affects the interactivities between blood Cr and liver steatosis is still unclear. The impact of blood Cr on hepatic steatosis in smokers requires further investigation.

Nevertheless, there are several limitations in our research. Firstly, because the gold standard for diagnosing hepatic steatosis—liver biopsy data weren't available, we evaluated the hepatic steatosis using CAP from liver ultrasound transient elastography. Secondly, there are still knowledge gaps and lack of international consensus on how to define Cr deficiency and toxicity. Referring to the definitive reference for clinical chemistry, we defined Cr deficiency as blood Cr below 0.7 μg/L (10). Thirdly, it is difficult to avoid recall bias caused by self-reported data obtained from questionnaires. Fourthly, NHANES database lacked the information of genetic variations, complete medical history and dietary intake, thus we cannot rule out the bias caused by the aforementioned confounding factors. Fifthly, the NHANES only examined blood Cr levels in individuals aged 40 and above, and there is a lack of blood Cr investigation in individuals below the age of 40, particularly in children and pregnant women. A clinical study reported that blood Cr levels relieved the degree of insulin resistance in obese children, and improved other characteristic pathogenic events, such as inflammation, oxidative stress, abnormal glucose metabolism and dyslipidemia (43). For maternal rat, chronic maternal Cr restriction in rats increased offspring body adiposity by programming their epigenome (44). Studies have shown that Cr enhances insulin resistance and ameliorates obesity in kids, pregnant women and their offspring. However, there is a lack of research on the association between blood Cr levels and hepatic steatosis in children and pregnant women, and further studies are required. Finally, as a cross-sectional study, the causality and temporal order between blood Cr and hepatic steatosis could not be clarified. Hence, prospective cohort studies with sufficient observation periods and sample sizes are desired in the future.

## 5 Conclusions

Our study demonstrates that Cr deficiency is widespread in the American population, and there is an independent negative correlation between blood Cr level and hepatic steatosis. These results suggest that elevating blood Cr by diary supplement or other manners may be a potential therapeutic strategy of FLD.

## Data availability statement

The original contributions presented in the study are included in the article/Supplementary material, further inquiries can be directed to the corresponding author.

## Ethics statement

This study was reviewed and approved by NCHS Ethics Review Board, and informed consent was obtained from all participants involved in the study.

## Author contributions

YXi: Writing—original draft, Validation, Methodology, Investigation, Funding acquisition, Formal analysis, Data curation, Conceptualization. RZ: Writing—original draft, Methodology, Data curation. ZW: Writing—original draft, Investigation. YXu: Writing—original draft, Investigation. YC: Writing—original draft, Investigation. LS: Writing—original draft, Investigation. ZZ: Writing—original draft, Investigation. PX: Writing—original draft, Investigation. GZ: Writing—original draft, Investigation. WS: Writing—review & editing, Validation, Supervision, Project administration, Funding acquisition, Data curation, Conceptualization.
