# Supplementary material for: Association between blood chromium and hepatic steatosis assessed by liver ultrasound transient elastography: National Health and Nutrition Examination Survey 2017–2020
Source: Front Nutr. 2024 Apr 24;11:1307519. doi: 10.3389/fnut.2024.1307519 (PMC11076870; doi:10.3389/fnut.2024.1307519)
Supplement: Supplementary file 1 [file Table_1.docx]

Supplementary Material

**Table S1. Baseline characteristics of the study population based on gender.**

| **Characteristic** | **Total**  **(N =4926)** | **Man**  **(N=2448)** | **Woman**  **(N=2478)** | ***p*-value** |  |  |
| --- | --- | --- | --- | --- | --- | --- |
| **Socio-demographic Characteristics** | | | |  |  |  |
| Age (years) | 59.79 ± 11.63 | 60.13 ± 11.76 | 59.46 ± 11.49 | <0.05 |  |  |
| Ethnicity | | | | 0.07 |  |  |
| Hispanic | 1047 (21.25%) | 490 (20.02%) | 557 (22.48%) |  |  |  |
| non-Hispanic White | 1787 (36.28%) | 913 (37.30%) | 874 (35.27%) |  |  |  |
| non-Hispanic Black | 1278 (25.94%) | 641 (26.18%) | 637 (25.71%) |  |  |  |
| non-Hispanic Asian | 597 (12.12%) | 284 (11.60%) | 313 (12.63%) |  |  |  |
| Other race | 217 (4.41%) | 120 (4.90%) | 97 (3.91%) |  |  |  |
| Education | | | | 0.12 |  |  |
| Less than high school | 983 (19.96%) | 520 (21.24%) | 463 (18.68%) |  |  |  |
| High school | 1174 (23.83%) | 585 (23.90%) | 589 (23.77%) |  |  |  |
| More than high school | 2760 (56.03%) | 1338 (54.66%) | 1422 (57.38%) |  |  |  |
| Not recorded | 9 (0.18%) | 5 (0.20%) | 4 (0.16%) |  |  |  |
| Family income-to-poverty threshold ratio (%) | | | | 0.65 |  |  |
| < 1.0 | 712 (14.45%) | 349 (14.26%) | 363 (14.65%) |  |  |  |
| 1.0 to < 2.0 | 1103 (22.39%) | 529 (21.61%) | 574 (23.16%) |  |  |  |
| 2.0 to < 3.0 | 677 (13.74%) | 331 (13.52%) | 346 (13.96%) |  |  |  |
| 3.0 to < 5.0 | 877 (17.80%) | 442 (18.06%) | 435 (17.55%) |  |  |  |
| ≥ 5 | 901 (18.29%) | 465 (19.00%) | 436 (17.59%) |  |  |  |
| Not recorded | 656 (13.32%) | 332 (13.56%) | 324 (13.08%) |  |  |  |
| **Physical examinations** | | | |  |  |  |
| LSM (kPa) | 5.10 (4.20-6.40) | 5.30 (4.40-6.60) | 4.90 (4.00-6.10) | <0.01 |  |  |
| CAP (dB/m) | 272.66 ± 60.21 | 278.79 ± 61.58 | 266.60 ± 58.20 | <0.01 |  |  |
| Weight (kg) | 82.88 ± 21.01 | 87.80 ± 19.88 | 78.01 ± 20.97 | <0.01 |  |  |
| BMI (kg/m^2^) | 29.93 ± 6.78 | 29.20 ± 5.70 | 30.65 ± 7.62 | <0.01 |  |  |
| **Biochemical indicators** | | | |  |  |  |
| ALT (U/L) | 18.00 (13.00-26.00) | 21.00 (16.00-29.00) | 16.00 (12.00-22.00) | <0.01 |  |  |
| AST(U/L) | 19.00 (16.00-24.00) | 21.00 (17.00-25.00) | 18.00 (15.00-22.00) | <0.01 |  |  |
| GGT (IU/L) | 22.00 (15.25-34.00) | 25.00 (18.00-41.00) | 19.00 (14.00-28.00) | <0.01 |  |  |
| ALP (IU/L) | 80.13 ± 25.72 | 78.09 ± 25.36 | 82.14 ± 25.93 | <0.01 |  |  |
| Total bilirubin (mg/dL) | 0.40 (0.30-0.60) | 0.50 (0.30-0.60) | 0.30 (0.30-0.50) | <0.01 |  |  |
| Urine albumin(ug/mL) | 9.40 (4.70-20.55) | 9.90 (5.12-23.28) | 8.80 (4.20-19.20) | <0.01 |  |  |
| Total calcium (mg/dL) | 9.28 ± 0.39 | 9.26 ± 0.37 | 9.29 ± 0.40 | <0.05 |  |  |
| TG (mg/dL) | 97.00 (67.00-139.00) | 99.00 (68.00-143.00) | 95.00 (67.00-136.00) | 0.05 |  |  |
| TC (mg/dL) | 190.07 ± 42.21 | 183.07 ± 42.62 | 197.03 ± 40.64 | <0.01 |  |  |
| LDL-C(mg/dL) | 111.42 ± 36.97 | 108.68 ± 36.94 | 114.12 ± 36.81 | <0.01 |  |  |
| HDL-C(mg/dL) | 54.09 ± 16.41 | 49.07 ± 14.10 | 59.08 ± 17.02 | <0.01 |  |  |
| Blood cobalt(ug/L) | 0.14 (0.11-0.18) | 0.13 (0.09-0.16) | 0.15 (0.12-0.20) | <0.01 |  |  |
| Blood chromium(μg/L) | 0.29 (0.29-0.29) | 0.29 (0.29-0.29) | 0.29 (0.29-0.29) | 0.66 |  |  |
| hs-CRP (mg/L) | 2.04 (0.91-4.44) | 1.73 (0.82-3.80) | 2.45 (1.03-5.37) | <0.01 |  |  |
| HbA1c (%) | 6.04 ± 1.18 | 6.09 ± 1.22 | 6.00 ± 1.14 | <0.01 |  |  |
| Insulin (μU/mL) | 10.11 (6.30-16.39) | 9.96 (6.21-16.47) | 10.28 (6.38-16.27) | 0.55 |  |  |
| HOMA-IR | 2.76 (1.66-4.88) | 2.84 (1.68-5.09) | 2.72 (1.62-4.72) | 0.35 |  |  |
| HOMA-IS | 0.11 (0.07-0.18) | 0.11 (0.07-0.17) | 0.12 (0.08-0.18) | <0.05 |  |  |
| eGFR(ml/min/1.73 m^2) | 79.87 ± 31.84 | 81.70 ± 29.71 | 78.08 ± 33.73 | <0.01 |  |  |
| **Lifestyle factors** | | | |  |  |  |
| Alcohol consumption | | | | <0.01 |  |  |
| Never | 1337 (27.14%) | 706 (28.84%) | 631 (25.46%) |  |  |  |
| ≤ 3 times/month | 2028 (41.17%) | 945 (38.60%) | 1083 (43.70%) |  |  |  |
| Once a week | 306 (6.21%) | 195 (7.97%) | 111 (4.48%) |  |  |  |
| ≥ 2 times/week | 583 (11.84%) | 386 (15.77%) | 197 (7.95%) |  |  |  |
| Not recorded | 672 (13.64%) | 216 (8.82%) | 456 (18.40%) |  |  |  |
| Smoking status | | | | <0.01 |  |  |
| Yes | 829 (16.83%) | 484 (19.77%) | 345 (13.92%) |  |  |  |
| No | 1377 (27.95%) | 855 (34.93%) | 522 (21.07%) |  |  |  |
| Not recorded | 2720 (55.22%) | 1109 (45.30%) | 1611 (65.01%) |  |  |  |
| **Comorbidities** | | | |  |  |  |
| Diabetes |  |  |  | <0.01 |  |  |
| Normal | 1944 (39.46%) | 919 (37.54%) | 1025 (41.36%) |  |  |  |
| Prediabetes | 1767 (35.87%) | 866 (35.38%) | 901 (36.36%) |  |  |  |
| Diabetes | 1215 (24.67%) | 663 (27.08%) | 552 (22.28%) |  |  |  |
| Hypertension |  |  |  | 0.78 |  |  |
| Normal | 2513 (51.02%) | 1258 (51.47%) | 1255 (50.71%) |  |  |  |
| Hypertension | 2406 (48.84%) | 1186 (48.53%) | 1220 (49.29%) |  |  |  |
| Not recorded | 7 (0.14%) | 4 (0.16%) | 3 (0.12%) |  |  |  |
| Hepatitis B | | | | <0.01 |  |  |
| Normal | 4403 (89.38%) | 2149 (87.79%) | 2254 (90.96%) |  |  |  |
| Hepatitis B | 521 (10.58%) | 297 (12.13%) | 224 (9.04%) |  |  |  |
| Not recorded | 2 (0.04%) | 2 (0.08%) | 0 (0.00%) |  |  |  |
| Hepatitis C | | | | <0.01 |  |  |
| Normal | 4742 (96.26%) | 2327 (95.06%) | 2415 (97.46%) |  |  |  |
| Hepatitis C | 183 (3.71%) | 120 (4.90%) | 63 (2.54%) |  |  |  |
| Not recorded | 1 (0.02%) | 1 (0.04%) | 0 (0.00%) |  |  |  |
| Autoimmune hepatitis | | | | 0.98 |  |  |
| Autoimmune hepatitis | 14 (0.28%) | 7 (0.29%) | 7 (0.28%) |  |  |  |
| Not recorded | 4912 (99.72%) | 2441 (99.71%) | 2471 (99.72%) |  |  |  |

Abbreviations: LDL-C: low-density lipoprotein cholesterol; HDL-C: high-density lipoprotein cholesterol; TC: total cholesterol; TG: triglyceride; HbA1c: glycated hemoglobin; hs-CRP: high-sensitivity C-reactive protein; ALT: alanine aminotransferase; ALP: alkaline phosphatase; AST: aspartate aminotransferase; GGT: gamma-glutamyl transferase; BMI: body mass index; LSM: liver stiffness measure; CAP: controlled attenuation parameter.

**Table S2. Distribution of blood chromium by gender, ethnicity, age, and fatty liver disease.**

| **Characteristic** | | **Chromium** | | **Total** |
| --- | --- | --- | --- | --- |
|  |  | **Deficiency(<0.7 μg/L)** | **Normal(≥0.7 μg/L)** |  |
| Total | | 4615(93.69%) | 311(6.31%) | 4926 |
| Gender | Female | 2329(93.99%) | 149(6.01%) | 2478 |
|  | Male | 2286(93.38%) | 162(6.62%) | 2448 |
| Ethnicity | Hispanic | 991(94.65%) | 56(5.35%) | 1047 |
|  | non-Hispanic Asian | 565(94.64%) | 32(5.36%) | 597 |
|  | non-Hispanic Black | 1189(93.04%) | 89(6.96%) | 1278 |
|  | non-Hispanic White | 1662(93.01%) | 125(6.99%) | 1787 |
|  | Other race | 208(95.85%) | 9(4.15%) | 217 |
| Age(years) | 40 to < 60 | 2266(94.69%) | 127(5.31%) | 2393 |
|  | 60-80 | 2349(92.74%) | 184(7.26%) | 2533 |
| Hepatic Steatosis | Hepatic Steatosis | 1949(94.43%) | 115(5.57%) | 2064 |
|  | non-Hepatic Steatosis | 2666(93.15%) | 196(6.85%) | 2862 |

Table S3. Relationship between blood chromium levels and the controlled attenuation parameter (CAP) after multiple interpolations.

| **Outcome** | **IMP 1** | | **IMP 2** | | **IMP 3** | | **IMP 4** | | **IMP 5** | | **Estimate from multiple imputed data** | |
| --- | --- | --- | --- | --- | --- | --- | --- | --- | --- | --- | --- | --- |
|  | **β(95%CI)** | ***p*-value** | **β(95%CI)** | ***p*-value** | **β(95%CI)** | ***p*-value** | **β(95%CI)** | ***p*-value** | **β(95%CI)** | ***p*-value** | **β(95%CI)** | ***p*-value** |
| **Model Ⅰ** | | | | | | | | | | | | |
| **Blood chromium** | -5.19 (-8.93, -1.46) | <0.01 | -5.19 (-8.93, -1.46) | <0.01 | -5.19 (-8.93, -1.46) | <0.01 | -5.19 (-8.93, -1.46) | <0.01 | -5.19 (-8.93, -1.46) | <0.01 | -5.19(-8.92, -1.46) | <0.01 |
| **Blood chromium categories** | | | | | | | | | | | | |
| **< 0.41μg/L** | Reference | | Reference | | Reference | | Reference | | Reference | | Reference | |
| **≥ 0.41, < 0.7 μg/L** | -4.78(-10.11, 0.55) | 0.08 | -4.78(-10.11, 0.55) | 0.08 | -4.78(-10.11, 0.55) | 0.08 | -4.78(-10.11, 0.55) | 0.08 | -4.78(-10.11, 0.55) | 0.08 | -4.78(-10.11, 0.55) | 0.08 |
| **≥ 0.7 μg/L** | -10.05 (-16.98, -3.11) | <0.01 | -10.05 (-16.98, -3.11) | <0.01 | -10.05 (-16.98, -3.11) | <0.01 | -10.05 (-16.98, -3.11) | <0.01 | -10.05 (-16.98, -3.11) | <0.01 | -10.05 (-16.98, -3.11) | <0.01 |
| ***p* for trend** | <0.01 | | <0.01 | | <0.01 | | <0.01 | | <0.01 | | <0.01 | |
| **Model Ⅱ** | | | | | | | | | | | | |
| **Blood chromium** | -4.83 (-8.52, -1.14) | <0.05 | -4.83 (-8.52, -1.14) | <0.05 | -4.83 (-8.52, -1.14) | <0.05 | -4.83 (-8.52, -1.14) | <0.05 | -4.83 (-8.52, -1.14) | <0.05 | -4.83(-8.52, -1.14) | <0.05 |
| **Blood chromium categories** | | | | | | | | | | | | |
| **< 0.41μg/L** | Reference | | Reference | | Reference | | Reference | | Reference | | Reference | |
| **≥ 0.41, < 0.7 μg/L** | -3.81(-9.09, 1.46) | 0.16 | -3.81(-9.09, 1.46) | 0.16 | -3.81(-9.09, 1.46) | 0.16 | -3.81(-9.09, 1.46) | 0.16 | -3.81(-9.09, 1.46) | 0.16 | -3.81(-9.09, 1.46) | 0.16 |
| **≥ 0.7 μg/L** | -9.32(-16.18, -2.46) | <0.01 | -9.32(-16.18, -2.46) | <0.01 | -9.32(-16.18, -2.46) | <0.01 | -9.32(-16.18, -2.46) | <0.01 | -9.32(-16.18, -2.46) | <0.01 | -9.32(-16.18, -2.46) | <0.01 |
| ***p* for trend** | <0.01 | | <0.01 | | <0.01 | | <0.01 | | <0.01 | | <0.01 | |
| **Model Ⅲ** | | | | | | | | | | | | |
| **Blood chromium** | -4.09 (-7.03, -0.87) | <0.05 | -4.02 (-7.23, -0.80) | <0.05 | -4.04 (-7.26, -0.83) | <0.05 | -3.98 (-7.19, -0.77) | <0.05 | -4.04 (-7.26, -0.83) | <0.05 | -4.02 (-7.23, -0.81) | <0.05 |
| **Blood chromium categories** | | | | | | | | | | | | |
| **< 0.41μg/L** | Reference | | Reference | | Reference | | Reference | | Reference | | Reference | |
| **≥ 0.41, < 0.7 μg/L** | -3.10(-7.33, 1.14) | 0.15 | -3.16(-7.40, 1.07) | 0.14 | -3.09(-7.32, 1.14) | 0.15 | -3.16(-7.39, 1.08) | 0.14 | -3.16(-7.39, 1.07) | 0.14 | -3.14 (-7.38, 1.09) | 0.15 |
| **≥ 0.7 μg/L** | -8.26(-13.81, -2.71) | <0.01 | -8.11(-13.66, -2.56) | <0.01 | -8.30(-13.85, -2.75) | <0.01 | -8.20(-13.74, -2.65) | <0.01 | -8.21(-13.76, -2.66) | <0.01 | -8.21(-13.76, -2.65) | <0.01 |
| ***p* for trend** | <0.01 | | <0.01 | | <0.01 | | <0.01 | | <0.01 | | <0.01 | |

Model Ⅰ: no covariates were adjusted.

Model Ⅱ: age, gender, race/ethnicity were adjusted.

Model Ⅲ: age, gender, race/ethnicity, education, family income-to-poverty threshold ratio, alcohol consumption, smoke, BMI, weight, TG, TC, LDL-C, HDL-C, insulin, HbA1c, HOMA-IR, HOMA-IS, hs-CRP, ALT, AST, GGT, ALP, total bilirubin, blood cobalt, total calcium, urine albumin, eGFR, hypertension, diabetes, hepatitis B, hepatitis C, autoimmune hepatitis, LSM were adjusted.
